# Parallel Regulation of Memory and Emotion Supports the Suppression of Intrusive Memories

**DOI:** 10.1523/JNEUROSCI.2732-16.2017

**Published:** 2017-07-05

**Authors:** Pierre Gagnepain, Justin Hulbert, Michael C. Anderson

**Affiliations:** ^1^Normandie Univ, UNICAEN, PSL Research University, EPHE, INSERM, U1077, CHU de Caen, Neuropsychologie et Imagerie de la Mémoire Humaine, 14000 Caen, France,; ^2^Bard College, Annandale-on-Hudson, New York 12504,; ^3^MRC Cognition and Brain Sciences Unit, University of Cambridge, Cambridge CB27EF, United Kingdom, and; ^4^University of Cambridge, Behavioural and Clinical Neuroscience Institute, Cambridge CB23EB, United Kingdom

**Keywords:** affect regulation, dynamic causal modeling, emotion, forgetting, inhibitory control, memory suppression

## Abstract

Intrusive memories often take the form of distressing images that emerge into a person's awareness, unbidden. A fundamental goal of clinical neuroscience is to understand the mechanisms allowing people to control these memory intrusions and reduce their emotional impact. Mnemonic control engages a right frontoparietal network that interrupts episodic retrieval by modulating hippocampal activity; less is known, however, about how this mechanism contributes to affect regulation. Here we report evidence in humans (males and females) that stopping episodic retrieval to suppress an unpleasant image triggers parallel inhibition of mnemonic and emotional content. Using fMRI, we found that regulation of both mnemonic and emotional content was driven by a shared frontoparietal inhibitory network and was predicted by a common profile of medial temporal lobe downregulation involving the anterior hippocampus and the amygdala. Critically, effective connectivity analysis confirmed that reduced amygdala activity was not merely an indirect consequence of hippocampal suppression; rather, both the hippocampus and the amygdala were targeted by a top-down inhibitory control signal originating from the dorsolateral prefrontal cortex. This negative coupling was greater when unwanted memories intruded into awareness and needed to be purged. Together, these findings support the broad principle that retrieval suppression is achieved by regulating hippocampal processes in tandem with domain-specific brain regions involved in reinstating specific content, in an activity-dependent fashion.

**SIGNIFICANCE STATEMENT** Upsetting events sometimes trigger intrusive images that cause distress and that may contribute to psychiatric disorders. People often respond to intrusions by suppressing their retrieval, excluding them from awareness. Here we examined whether suppressing aversive images might also alter emotional responses to them, and the mechanisms underlying such changes. We found that the better people were at suppressing intrusions, the more it reduced their emotional responses to suppressed images. These dual effects on memory and emotion originated from a common right prefrontal cortical mechanism that downregulated the hippocampus and amygdala in parallel. Thus, suppressing intrusions affected emotional content. Importantly, participants who did not suppress intrusions well showed increased negative affect, suggesting that suppression deficits render people vulnerable to psychiatric disorders.

## Introduction

Sometimes the past intrudes upon the present. Although a passing disturbance for most people, such intrusive memories can be vivid, persistent, and distressing for individuals suffering from post-traumatic stress, anxiety, or obsessive-compulsive disorders (Brewin et al., 2010). Indeed, distressing images are thought to both precipitate psychopathological symptoms and contribute to their maintenance (e.g., Rachman, 2007; Brewin et al., 2010; Moritz et al., 2014). Understanding why some people have difficulty controlling memories requires that we characterize the neural systems that inhibit memory intrusions and that attenuate the distress they cause. Here we examine how people suppress the retrieval of intrusive images, focusing on whether and how this process contributes to regulating affect.

Despite differing goals, memory control and affect regulation engage similar brain regions. For example, suppressing retrieval engages the right middle frontal gyrus (MFG) and reduces retrieval-related hippocampal activity (Anderson et al., 2004; Depue et al., 2007; for review, see Anderson and Hanslmayr, 2014; Anderson et al., 2016). Effective connectivity analyses indicate that these reductions arise from inhibitory modulation by the right MFG (Benoit and Anderson, 2012; Gagnepain et al., 2014) that increase forgetting of suppressed traces and reduce their tendency to intrude involuntarily (Benoit et al., 2015). Similarly, regulating emotional responses to negative stimuli engages right MFG (Ochsner et al., 2004; Eippert et al., 2007; Kim and Hamann, 2007; Hayes et al., 2010) to suppress emotion-related activity in the amygdala (Kohn et al., 2014; Radaelli et al., 2015; Comte et al., 2016). Comparisons of retrieval suppression and affect regulation confirm their overlapping localization within the right MFG (Depue et al., 2016). Critically, suppressing retrieval of aversive images without affect regulation instructions reduces hippocampal and amygdala activity (Depue et al., 2007, 2010), suggesting that suppression regulates memory and affect during unpleasant intrusions.

**Figure 1.**
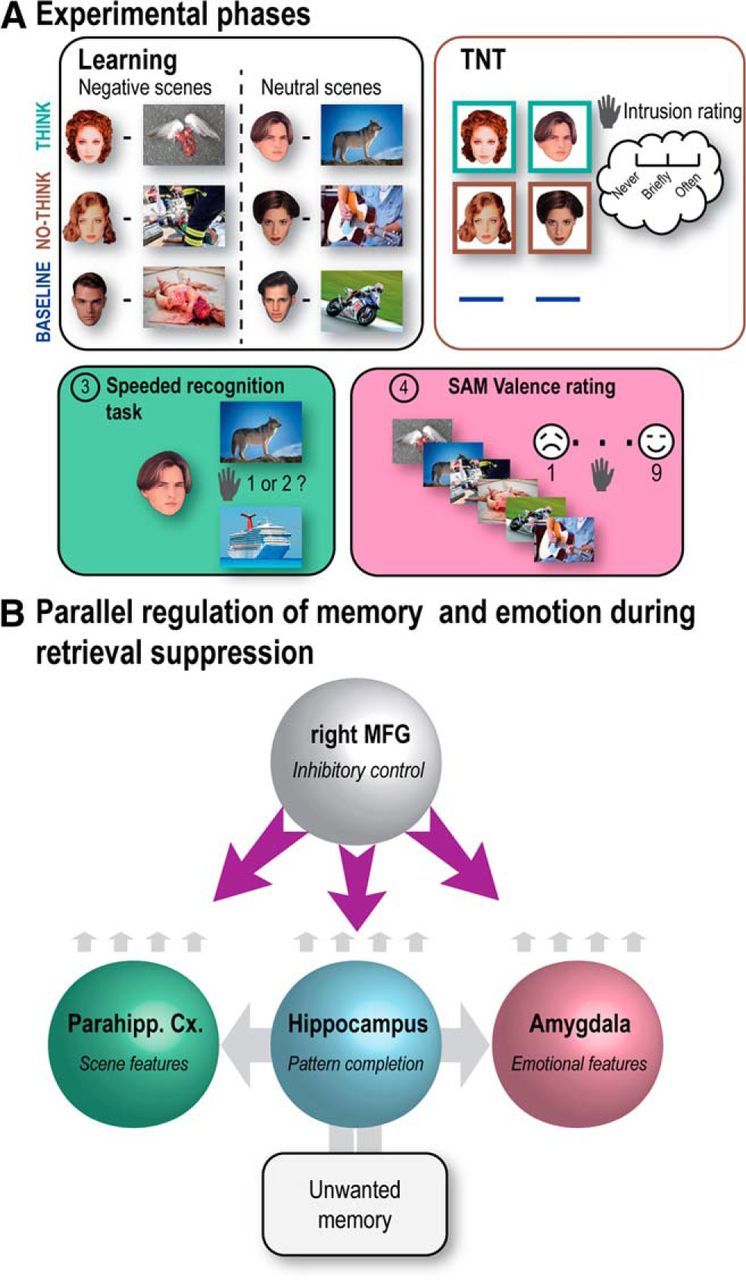
Experimental phases and hypothesized dynamics. ***A***, After learning face-scene pairs (Negative or Neutral), participants were scanned during the TNT task. For Think items (bounded by green box), participants recalled the associated scene; for No-Think items (bounded by red box), they tried to stop the memory of the scene from entering awareness. Baseline cues were not presented during this TNT phase. Next, participants performed a speeded associative recognition task followed by an SAM valence rating task on all picture categories (Think, No-Think, and Baseline) to evaluate how suppression affected memory and emotional perceptions, respectively. On these final tests, baseline items provided an estimate of memory or affect, given that neither retrieval nor suppression has been performed in the interim. The pictures displayed here are not issued from IAPS database but are free-use pictures taken from the internet for illustrative purpose. ***B***, The dynamics predicted to bring about parallel inhibition of memory and emotion for intrusive negative scenes. Cue input to the hippocampus is predicted to drive pattern completion, followed by recurrent reactivation of scene and emotional features in parahippocampal cortex and amygdala, respectively; intrusion-related reactivation in these regions is predicted to trigger parallel inhibition by the right MFG. We do not propose that MFG directly inhibits these structures given the weak anatomical projections between MFG and amygdala (Anderson et al., 2016). However, the MFG is proposed to modulate these regions polysynaptically via pathways yet to be fully understood.

How retrieval suppression might regulate negative affect is unclear. One possibility is that suppression downregulates hippocampal activity, preventing reinstatement of upsetting imagery; this may truncate input into the amygdala, reducing its activity and preempting emotional responses to the memory. Alternatively, suppression may inhibit both hippocampal and amygdala processes, rendering unpleasant memories less intrusive and upsetting. This form of parallel modulation has precedent. For example, effective connectivity analyses show that right MFG inhibits both hippocampal and fusiform cortex activity when participants suppress memories of visual objects (Gagnepain et al., 2014); this modulation predicts reduced priming in fusiform cortex on later perceptual identification tests, indicating that the objects' sensory representations were suppressed. Gagnepain et al. (2014) argued that retrieval cues had triggered intrusions, driving reentrant signals from the hippocampus to reinstate the objects' sensory features. Suppressing object memories might therefore have engaged inhibitory control targeted at both hippocampus and visual cortex. Analogously, suppressing emotional images may trigger inhibitory processes targeted at emotion and scene features reinstated during intrusions (Fig. 1*A*) (Gagnepain et al., 2014).

To test this parallel modulation hypothesis, we conducted fMRI as participants suppressed episodic retrieval. Participants performed the Think/No-Think (TNT) task (Anderson and Green, 2001), which included trials requiring them to attend to a reminder (a face) of a scene that was either aversive or neutral; for each reminder, they were cued to retrieve the scene (Think items) or to suppress its retrieval (No-Think items) (Fig. 1*B*). After each trial, participants classified whether the reminder elicited awareness of its paired scene (Levy and Anderson, 2012), allowing us to isolate when No-Think trials triggered intrusions. After this phase, participants rated the Think and No-Think scenes' valence, along with previously studied Baseline scenes not presented during this TNT phase. We tested three predictions using behavioral partial least-squares (PLS) (McIntosh and Lobaugh, 2004; Krishnan et al., 2011) and dynamic causal modeling (DCM) (Friston et al., 2003). First, we considered whether a common area within right MFG exists whose intrusion-related activations predict both better intrusion control and reduced negative affect for suppressed scenes. Second, we examined whether shared suppression-related deactivations in the hippocampus, parahippocampus, and amygdala predict both intrusion control and reduced affect. Third and critically, effective connectivity analyses should reveal that right MFG modulates the hippocampus, parahippocampus, and amygdala in parallel, and that intrusions trigger greater negative coupling.

## Materials and Methods

### 

#### Experimental design

##### Participants.

Twenty-four right-handed native English speakers between the ages of 18 and 35 years were paid to participate (8 males). They had no reported history of neurological, medical, visual, or memory disorders. The project was approved by both the University of Oregon Institutional Review Board and Cambridge Psychology Research Ethics Committee, and all participants gave written consent. Participants were asked not to consume psychostimulants, drugs, or alcohol before the experimental period. Two participants were excluded given that they had an insufficient number of intrusions, <5% (i.e., 4 trials) within a given emotional condition (see below), for the purposes of fMRI analyses.

##### Material.

The stimuli were 48 face-scene pairs plus 10 filler pairs selected from the International Affective Picture System (IAPS) (Lang et al., 2008) database. Half of the critical scenes were normed as negative (IAPS number: 1301, 2053, 2141, 2700, 2710, 2900, 3280, 6020, 6244, 6571, 6831, 9041, 9042, 9102, 9180, 9181, 9320, 9420, 9470, 9520, 9561, 9800, 9830, 9911; mean valence = 2.8; SD valence = 1.7; mean arousal = 5.3; SD arousal = 2.3), whereas the other half were neutral (IAPS number: 1121, 1313, 1640, 1810, 2250, 2487, 2616, 4100, 4535, 5395, 5455, 5628, 7289, 7351, 7402, 7480, 7495, 7503, 7510, 7560, 7640, 8060, 8117, 8250; mean valence = 5.9; SD valence = 1.7; mean arousal = 4.8; SD arousal = 2.2). Three lists of 8 pairs (assigned to Think, No-Think, and Baseline conditions) were created for each valence condition and were counterbalanced so that they appeared in each TNT condition equally often, across participants.

##### Procedure.

To match the strength of initial encoding for all pairs, participants first learned all face-scene pairs through a drop-off/feedback cycle procedure. After studying all pairs for 6 s each, participants were given test trials presenting the face cue for a given pair for up to 4 s and asked whether they could recall and fully visualize the paired scene. If so, three scenes then appeared (one correct and two foils taken from other pairs), and they received up to 5 s to select which scene went with the face cue. After selecting a scene or if the response window expired, a screen appeared for 1 s indicating whether the recognition judgment was correct, incorrect, or was not registered before the end of the trial. In all cases (even if participants indicated that they could fully visualize the associated scene in the first step), each trial ended with the correct pairing appearing onscreen for 3.5 s. Participants were asked to use this feedback to increase their knowledge of the pair. Once all pairs had been presented, all of the face-scene pairs not recalled or correctly recognized were presented again in a randomized order until each pairing had been correctly identified once. After testing all pairs in this manner, a second drop-off/feedback cycle was applied in the same manner, thus ensuring accurate and strong memory for all pictures. This procedure ensured that all pairs were learned to a comparable degree and that any encoding advantage for negatively valenced stimuli was carefully controlled.

Following learning and after practice with the TNT task, participants entered the MRI scanner. At this point, participants engaged in a final round of TNT practice that was followed by a brief reminder of all of the studied pairs (1.5 s each), during which participants were asked once again to reinforce their knowledge of the pairings. This overtraining procedure was intended to ensure that images would intrude when its cue was presented during the TNT phase, allowing us to isolate brain regions engaged to control these intrusions.

Participants then performed the TNT task, which was divided into 5 sessions, each 7–8 min in length. Each session presented two repetitions of 16 Think (8 Negative and 8 Neutral face-cues) and 16 No-Think (8 Negative and 8 Neutral face-cues) items, yielding, across the 5 sessions, 160 trials per condition in total (32 trials × 5 sessions). Cues appeared for 3 s either framed in green or red, centered on a black background. On Think trials, the cue was bounded by a green box, and participants were told to generate as detailed and complete an image of the associated scene as possible. On No-Think trials, the cue was bounded by a red box, and participants were told that it was imperative to prevent the scene from coming to mind at all and that they should fixate and concentrate on the face-cue without looking away (they knew their eye movements were being monitored). During red-cued trials, participants were asked to block thoughts of the scene by blanking their mind and not by replacing the scene with any other thoughts or mental images. If the object image came to mind anyway, they were asked to push it out of mind.

After the offset of each of the Think or No-Think trial cues, participants reported the extent to which the associated scene had entered awareness by pressing one of three buttons corresponding to the labels: never, briefly, often. Although participants had up to 10 s to make this rating, they were instructed and trained to make this rating quickly without thinking about the associated picture. Their response was followed by a jittered fixation cross lasting 500–8000 ms (mean ± SD, ∼2200 ± 2000 ms depending on sessions), optimized to increase the efficiency of the event-related response estimation. These “intrusion ratings” were used to isolate trials with intrusive memories and quantify their occurrence. Specifically, we used participants' responses to classify each trial as either having an intrusion (i.e., a “briefly” or “frequent” response) or not (a “never” response) in binary fashion. For each repetition of a given condition (e.g., the No-Think, negative-valence condition), we averaged these binary intrusion reports across all 8 items in that condition to compute an intrusion proportion for that repetition. We then averaged these intrusion proportions across the 10 repetitions of TNT instruction (i.e., across the 2 repetitions in each of the 5 sessions) to derive the overall intrusion rate for a given participant. At the end of the 5 TNT sessions, participants also performed a spatial cueing task for 8 min (data not reported here).

Outside the scanner, aftereffects of memory suppression were examined via an associative recognition memory test on Think and No-Think items. In addition, a third group of items (Baseline items) was tested during this task. These items had been trained in the same initial learning session with the Think and No-Think items but were omitted from the TNT phase. Because these items were trained at the same time as the Think and No-Think items, but did not participate in the TNT task, they provide a baseline estimate of memorability of the scenes, given that neither suppression nor retrieval had been performed on them. These pairs enabled us to assess the effects of retrieval and suppression on the retention of Think and No-Think items, respectively, against pairs that were similarly old. During trials of this recognition task, a single face cue was presented for 5000 ms along with a target and a single foil sampled from other pairs. We expected nearly perfect recognition accuracy on this test; thus, we focused on whether repeated suppression of scene memories slowed subsequent recognition. Accordingly, participants were instructed to make their response as quickly as they could. Following their response, a 4 point confidence scale appeared on the screen and participants had to indicate whether they were 1 (not confident) to 4 (highly confident) about their response. We chose to measure recognition memory in this procedure instead of using recall tests more typically used in retrieval suppression studies, out of concern that our overtraining regimen (used to ensure sufficient intrusions) would cause ceiling effects in recall. By measuring recognition, we nevertheless could calculate recognition speed for all items, providing a way to assess suppression-induced forgetting, despite ceiling effects.

In the final phase, participants were asked to rate a series of pictures using Self-Assessment-Manikin (SAM) pictorial scale (Lang et al., 2008) to measure whether retrieval suppression influenced later affective responses to scenes. During this task, we included not only the Think and No-Think items, but also the aforementioned Baseline items, to enable us to determine whether retrieval or suppression altered the valence of items, relative to Baseline pairs. This 9 point scale quantifies participants' self-reported emotional response to each visual scene, reported on a scale labeled with diagram-like manikins with varying emotional facial expressions. Participants selected the numbered facial expression most closely matching their perception of the valence of the scene. Because the SAM valence scale includes “neutral” ratings at mid-scale [with negative affect on the low end (score 1–4), and positive affect on the high end (scores 6–9)], higher valence scores for suppressed Negative scenes likely reflect diminished negative affect (e.g., moving from a 1 to a 3 involves a movement toward neutral); in contrast, higher valence scores for neutral scenes likely reflect increasing positive affect. Participants were instructed to press a number from 1 (corresponding to a frowning face on the far left of the scale) if a picture made them feel completely unhappy to 9 (corresponding to a smiley face on the far right if a picture made them feel completely happy). If participants felt neutral, neither happy nor sad, they were then instructed to press the 5 key under the figure in the middle.

We used the foregoing ratings to compute valence scores for each condition for each participant, adjusted for pre-experimental valence. This adjustment assumes that the SAM valence rating that a participant gives for a scene is likely to be influenced by three contributing components: the scene's initial valence + the effects of our experimental manipulation for that participant (here, the effects of the TNT task) + noise (e.g., attentional fluctuation). To account for a part of the unexplained variance and provide a better estimation of the impact of TNT manipulation, we expressed the SAM valence rating given by a particular subject for each item as a percentage relative to that item's IAPS normative value (Lang et al., 2008), which gives a sample-based estimate of the probable starting valence for that scene. We performed this correction because we lacked pre-experiment measures of scene valence that were specific to each participant, so that we could remove variance across conditions contributed by premanipulation item differences (above and beyond our attempts to match this during material selection), attenuating list-based bias. Ideally, future studies should compute this adjustment for starting valence using participant-specific ratings of the scenes collected before the TNT phase, rather than sample-based normative values.

Outlier trimming was applied at the item level to recognition reaction times and adjusted SAM valence rating data, with outliers defined as >2× above or below median absolute deviation (MAD) (Leys et al., 2013) computed separately for each condition. MAD is a robust measure of dispersion given by the following formula:

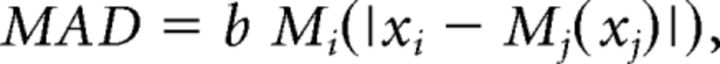
 where *x*_j_ is the *n* original observations, *M_i_* the median, and *b* = 1.4826, a constant linked to the assumption of normality of the data, disregarding the abnormality induced by outliers (Leys et al., 2013). Details of statistical analyses for behavioral data can be found in Results.

#### Imaging acquisition parameters

Scanning was performed on a 3-T Siemens Allegra MRI system at the Lewis Center for Neuroimaging at the University of Oregon using a 32-channel whole-head coil. High-resolution (0.5 × 0.5 × 1 mm) T1-weighted image was collected for anatomical visualization and normalization. Functional data were acquired using a gradient-echo, echo-planar pulse sequence (repetition time = 2000 ms, echo time = 30 ms, 31 interleaved slice acquisition, 3 × 3 × 3 mm voxel size). The first eight volumes of each session were discarded to allow for magnetic field stabilization.

#### fMRI preprocessing

Data were analyzed using Statistical Parametric Mapping software (SPM12, Wellcome Department of Imaging Neuroscience, London; RRID:SCR_007037). During preprocessing, images were first corrected for slice acquisition temporal delay before being spatially realigned to correct for motion. Images were then normalized using the parameters derived from the nonlinear normalization of individual gray-matter T1 images to the T1 template of the MNI and spatially smoothed using a 10 mm FWHM Gaussian kernel for second-level univariate analyses. However, native space 4 mm smoothed images were used for medial temporal lobe (MTL) region of interest (ROI) analyses to ensure maximum accuracy and demarcation between MTL hand-drawn ROIs (i.e., parahippocampal cortex, hippocampus, and amygdala).

#### ROI definition for activation, brain-behavior correlation, and DCM analyses

We defined an identical set of ROIs across activation, brain-behavior correlation, and DCM (Friston et al., 2003) analyses as follows. Given the fine-grain demarcation between the amygdala and the hippocampus, warping during the image normalization stage may introduce spatial errors, mixing up voxels from these structures. To address this issue, we first made hand-drawn, participant-specific MTL masks, based on the individual T1-weighted image. Anatomical demarcation was done according to Frankó et al. (2014) and Pruessner et al. (2000, 2002).

Within these participant-specific native MTL masks, the maximum peak was identified using the No-Think < Think contrast. Then, from this peak, an in-house program was used to select the most significant contiguous voxels corresponding to 10% of the total mask size. A new mask was created from these voxels with an average volume across participants of 200, 245, and 160 mm^3^ for the parahippocampal cortex, hippocampus, and amygdala, respectively. Selecting the voxels around the strongest peak is important for DCM analyses because effective connectivity analyses are meaningless in the absence of univariate effects. However, in this context, creating a sphere around the peak, as is often done, would not have been appropriate because such spheres could include voxels from different MTL structures. Our approach thus ensures that selected voxels exhibit a strong univariate effect between Think and No-Think conditions and also respect anatomical boundaries. Within this mask, we extracted parameter estimates using non-normalized images (i.e., participant's native space) to ensure maximum accuracy and demarcation between ROIs and computed the Intrusion versus Non-Intrusion contrast (for each valence condition). We note that the contrast used to select this mask (collapsing across all No-Think trials vs Think trials) is orthogonal to the comparison of Intrusion versus Non-Intrusion trials, thus avoiding circularity issues when we compared those conditions. Figure 3*B* reports the individual peak foci once projected back to the normalized MNI space for illustrative purpose.

We used a similar procedure to create an ROI that reflects activity relating to control. We focused on the anterior section of the right MFG, which encompasses the putative supramodal inhibitory control region spanning motor, memory, and emotion inhibition described by Depue et al. (2015). Given that this putative supramodal region lies in the anterior section of the right MFG, but that there are no clear anatomical boundaries to define it (unlike the hippocampus), we defined an initial binary mask based on the cluster centered alongside the right MFG from the group-level No-Think > Think contrast. We then restricted this mask to voxels with “*y*” coordinates above 30 mm in MNI space (corresponding to the anterior half of the MFG whose coordinates approximately range from 0 to 60 mm on the “*y*” axis). Two local maxima within this functional cluster are close to the supramodal region described by Depue et al. (2015) and were located in the following MNI coordinates: *x* = 30; *y* = 48; *z* = 16; and *x* = 28; *y* = 48; *z* = 32 (see Table 2). This MFG mask was then projected back into participants' native spaces using inversed normalization parameters. Within these participant-specific native masks, the individual peak maximum was identified using the No-Think > Think contrast, and the most significant contiguous voxels corresponding to 5% of the total mask size were selected (to account for the bigger initial mask volume compared with MTL mask). A new mask was created from these voxels with an average volume across participants of 460 mm^3^.

#### Statistical analyses of fMRI data

##### TNT univariate analyses.

The preprocessed time series in each voxel from the main TNT task was concatenated across sessions to facilitate subsequent DCM analyses. Regressors within a GLM for each voxel were created by convolving a boxcar function (modeled as a 3 s short epoch) at stimulus onset for each condition of interest (i.e., Think, Intrusion, and Non-Intrusion for both Negative and Neutral scenes) with a canonical HRF. In addition to the regressors of interest, further regressors of no interest were included, specifically the six realignment parameters, sines and cosines of up to three cycles per run to capture low-frequency drifts, and constant terms to remove the mean of each run. Filler items, along with the few items with no button press or not recalled during Think condition, were also entered into a single regressor of no interest. For ROI analyses (see ROI definition), individual parameter estimates were then extracted and averaged in each ROI. ROIs were analyzed using ANOVAs with Hemisphere, Region, Emotion, and Awareness (Intrusion vs Non-Intrusion) as within-subject factors for MTL, and Emotion and Awareness (Intrusion vs Non-Intrusion) as within-subject factors for the right MFG. Planned comparisons between experimental conditions of interest were performed using paired *t* test. Voxel-based analyses were also performed by entering first-level activation maps for each condition of interest into flexible analyses of variance (ANOVAs) implemented in SPM (RRID:SCR_007037), which used pooled error and correction for nonsphericity to create *t* statistics. The SPMs were thresholded for voxels whose statistic exceeded a peak threshold corresponding to *p* < 0.05 family-wise error (FWE) correction across the whole brain or within the appropriate search volumes of interest using random field theory.

##### Brain-behavior correlation analyses.

To compute brain-behavior relationships, we used a robust statistical approach based on the robust-correlation toolbox (Pernet et al., 2013). First, we rejected the null hypothesis based on the percentile bootstrap CI, an approach less sensitive to heteroscedasticity of the data than the traditional *t* test. Second, we corrected those bootstrapped CIs for multiple comparisons across our six MTL ROIs (left and right parahippocampal cortex, hippocampus, and amygdala), yielding a 99.3% CI. Third, we used skipped correlations (accounting for bivariate outliers using the S-estimator deviation rule) (Rousseeuw and Van Drissen, 1999), which estimate the true association with accurate false positive control and without loss of power.

For each ROI, we computed the Intrusion versus Non-Intrusion contrast and correlated this with both intrusion proportion (the number of No-Think trials for which an intrusion was reported divided by the number of No-Think trials across sessions) and affect suppression score (No-Think − Baseline adjusted rating).

##### Behavioral PLS correlation analyses.

ROI-behavior correlation analyses do not, however, allow us to ascertain whether the exact same voxels contribute to both mnemonic and affective regulation. Indeed, the hypothesis of a shared regulation mechanism across domains proposes that a shared set of voxels across the control network produces relationships for affect and memory suppression. However, ROI-behavior correlations could, in principle, be supported by different sets of voxels. Behavioral PLS correlation (McIntosh and Lobaugh, 2004; Krishnan et al., 2011) is ideally suited to disentangle this issue and examine more closely the relationship between neural markers of inhibition and behavioral scores across the putative memory control network (see below) and also MTL voxels. Behavioral PLS is a multivariate technique that reduces a set of voxels (i.e., variables) into a ranked series of independent latent variables (LVs) that express the largest possible covariance (or correlation) with behavioral scores. Put simply, this technique tries to identify separate sets of voxels that express quantitatively different relationships with behavioral measures. Voxel activity first has to be aligned and stacked across participants into a brain activation matrix *X* of 22 rows (i.e., participants) and *N* voxels. Normalized brain images are therefore used for that purpose. In a first series of PLS analyses, the mask used to create brain activity matrix *X* was extracted from the univariate analysis of No-Think > Think contrast (*p* < 0.001, uncorrected) and reflected memory control activity (i.e., frontoparietal control network). Within this mask, the Intrusion > Non-Intrusion contrast (for each type of emotional material) was computed for each voxel and the resulting vector of voxels was then stacked across participants. The same procedure was applied to the neural marker of downregulation in MTL (i.e., Non-Intrusion > Intrusion contrast). Given that this procedure requires the voxels to be aligned across participants, the MTL mask was derived by combining left and right parahippocampal cortex, hippocampus, and amygdala, defined anatomically using the AAL atlas (Tzourio-Mazoyer et al., 2002) (RRID:SCR_003550). The AAL atlas only includes a demarcation of the entire parahippocampal gyrus, which was then divided by splitting the mask into anterior (corresponding to perirhinal cortex) and posterior portions to isolate parahippocampal cortex.

Both intrusion proportion and affect suppression scores were entered into a *Y* matrix with participants representing rows. *Y* and *X* data tables were then mean-centered. Each participant vector of activation was additionally normalized such that the sum of squares of all its voxel values was equal to 1. This normalization ensured that the voxels of each participant's vector of activation now have the same variance and that differences between participants are not due to overall differences in activation (Abdi et al., 2012). The cross-block product of *X* and *Y* (i.e., *Y*^T^ × *X*) hence produced a matrix (R) encoding the relationship between each voxel and behavioral measurements across participants. A singular value decomposition was then applied to this R correlation matrix, such that R is decomposed into three matrices *r* = *U* × Δ × *V*^T^, with *U* being the matrix of behavioral saliences (i.e., weight), *V* voxel saliences, and Δ the amount of cross-table covariance accounted for by each LV. Singular value decomposition identifies the LVs that maximize the covariance between voxel activation (*X*) and behavioral measurements (*Y*). Each LV in *V* contains a spatial pattern depicting the brain regions where the activation shows the strongest relation to our behavioral scores. The brain scores (*X*^T^ × *V*) reflect the summary contribution of each participant's expression of a particular LV pattern. Correlations between participants' brain scores and behavioral variables thus indicate how each LV optimally represents relations between behavior and brain activity. The statistical significance of LVs was assessed using 5000 permutations in which participants' brain data matrices were randomly reassigned to behavioral measurements with the singular value recomputed each time. The number of times a singular value exceeds the observed singular value yields the probability of significance of original LVs. To compute the significance of voxel salience, we applied bootstrapping with replacement and recomputed singular value decomposition for each new bootstrap sample (5000 in total). This procedure yields a bootstrap distribution of voxel saliences which can then be transformed into a Bootstrapped Standard Ratio (BSR) equivalent to *z* score (by dividing initial voxel salience by the SE of the bootstrapped distribution) to assess the significance of a given voxel (McIntosh and Lobaugh, 2004). This multivariate technique quantifies the relationship between a voxel and a given dimension and is performed in a single analytic step, and thus does not require correction for multiple comparisons across voxels unlike if multiple univariate independent tests had been performed (McIntosh and Lobaugh, 2004).

##### TNT DCM analyses.

DCM explains changes in regional activity in terms of experimentally defined modulations (“modulatory input”) of the connectivity between regions. Here, we used DCM and Bayesian Model Selection (BMS) (Penny et al., 2010) to assess (1) whether right MFG suppresses MTL substructures during No-Think trials; (2) whether this modulation is targeted only at our emotion-related ROI (i.e., amygdala), only at our memory-related ROIs (i.e., parahippocampal cortex and hippocampus) or both memory and emotional regions; and (3) whether this inhibitory modulation increases in response to the elevated control demand arising during intrusions. DCM entails defining a network of a few ROIs and the forward and backward connections between them. The neural dynamics within this network are based on a set of simple differential equations (the bilinear state equation was used here) relating the activity in each region to (1) the activity of other regions via intrinsic connections in the absence of any experimental manipulation; (2) experimentally defined extrinsic input (or “driving input”) to one or more of the regions; and, most importantly, (3) experimentally defined modulations (or “modulatory input”) in the connectivity between regions. Changes in the network dynamics are caused by these driving (entering-regions) or modulatory (between-regions) inputs. These neural dynamics are then mapped to the fMRI time series using a biophysical model of the BOLD response. The neural (and hemodynamic) parameters of this DCM are estimated using approximate variational Bayesian techniques to maximize the free-energy bound on the Bayesian model evidence. Here, BMS was used to select the preferred model at the group level, treating the optimal model across participants as a random effect.

Retrieval inhibition was assumed to originate from right MFG (see Introduction, Results); therefore, we focused on the influence of this region over MTL regions within the same hemisphere as done in previous studies analyzing effective connectivity using the TNT paradigm (Benoit and Anderson, 2012; Benoit et al., 2015; Gagnepain et al., 2014; see also Benoit et al., 2016). To ensure that spurious relationships between MTL substructures were not captured by this analysis of effective connectivity, DCM was performed using participants' native space images smoothed at 4 mm to maximize demarcation between MTL ROIs (similar to MTL ROIs analysis; see ROI definition). For each ROI, the first eigenvariate was extracted and adjusted for effects of no interest (which included the six realignment parameters, sines and cosines of up to three cycles per run to capture low-frequency drifts, and constant terms to remove the mean of each run). The main goal of this analysis was to assess whether and how retrieval inhibition originating from right MFG was transmitted to MTL regions.

Thirty-five DCM models were created (for an illustration of the model space, see Fig. 7). All models had bidirectional connections between the parahippocampal cortex and hippocampus, the parahippocampal cortex and amygdala, and between the hippocampus and amygdala. A common input source across models driving network activity was defined as Think and No-Think trials entering the right MFG and are meant to represent the influence of memory control task instructions in the right MFG. These 35 models could be divided into two model families. The first family of models (the Modulatory family) divided the model space into five subgroups that differed according to whether the intrinsic connection from the right MFG was additionally modulated or not by No-Think items (modeled here as 3 s short epochs separately for Intrusion and Non-Intrusion of each emotion type). In the first subgroup, models included a bottom-up modulation during No-Think trials of the connection from the MTL to the MFG. In the second and third subgroups, this modulation was either top-down or bidirectional, respectively. In the fourth subgroup, modulation of MTL activity during No-Think trials was assumed to be driven by an afferent input originating from a source independent of the MFG. Finally, the fifth subgroup of models did not include any additional modulation during No-Think trials. The second family of models (the Regulation family) encodes the nature of emotional and memory regulation. In the first subgroup of this family (Emotion Regulation; see Fig. 7, first column), neural activity relating to emotion is directly targeted in the amygdala and memory suppression is an indirect consequence of downregulating amygdala activity, which is then echoed in other MTL regions. Therefore, this partition includes a single model in which the amygdala is targeted by MFG. The second subgroup represents models in which memory-related reactivation is directly targeted in the hippocampus and/or parahippocampal cortex and in which changes in emotion-related activity in the amygdala is an indirect consequence of memory suppression (Memory Regulation; see Fig. 7, middle column). Finally, the third partition is composed of models in which memory and emotion sites are targeted in parallel and therefore include a modulation combining parahippocampal cortex and/or hippocampus, and the amygdala (Parallel Regulation; see Fig. 7, right column). After estimating all 35 models for each participant, we performed the group BMS as implemented in SPM12 (version DCM12 revision 4750; RRID:SCR_007037). This produces the exceedance probability (i.e., the extent to which each model is more likely than any other model) and expected posterior probability (i.e., the probability of a model generating the observed data). We also tested whether coupling parameters differed significantly from zero using 5000 bootstrapping resamplings of the sum of intrinsic connections and modulatory parameters (i.e., DCM.A + DCM.B), and applying Bonferroni correction across tested parameters. Statistical differences between coupling parameters were assessed with an ANOVA using Region, Emotion, and Awareness as within-subject factors.

The foregoing model space does not model the full set of pathways that are likely involved in achieving mnemonic and affective regulation. For instance, other regions that are likely to be involved, based on prior studies, include the anterior cingulate cortex, presupplementary motor area, right inferior frontal gyrus, ventromedial prefrontal cortex, intraparietal cortex, and supramarginal gyrus (for review, see Anderson et al., 2016). We omitted these other regions for several reasons. First, it is not presently known which intermediate pathways are critical for modulating activity in the ROIs within the MTL. Second, the theoretical question to be addressed in the analysis did not concern these pathways, but rather whether MFG was involved in causally influencing mnemonic and affective processing in parallel, however that influence might be mediated. Third, the ability to estimate model parameters effectively is diminished the more complex models become. We therefore adopted the simplest model space that still allowed us to address our key theoretical questions about mnemonic and affective control. The winning model is therefore not intended to be a full anatomical specification of how mnemonic control is achieved, but rather a focused answer to a question about causal dynamics.

## Results

### No reliable differences in rate of learning were observed during training

During training, participants took 1.64 and 1.65 trials, on average, during the first dropoff training phase, to learn the negative and neutral face-scene associations, respectively. During the second dropoff training cycle, participants required 1 trial, on average (for both stimulus classes) to show evidence of having learned the associations. The maximum number of exposures was on average 3.62 and 3.64 for negative and neutral scenes, respectively, to reach the criterion of perfect performance across both cycles. No reliable differences in rate of learning were found.

### Success at suppressing intrusive memories and reducing negative affect of unpleasant experiences are related

**Figure 2.**
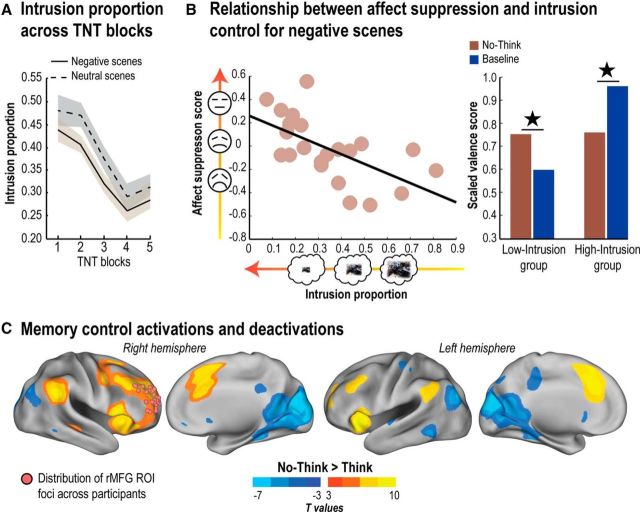
Behavioral and neural indices of mnemonic and affective regulation. ***A***, Intrusion proportions (i.e., the proportion of trials in which the associated memory entered into awareness on No-Think trials as measured by our trial-by-trial intrusion report measure; see Procedure) over the five scanning blocks of the TNT phase. Shaded error bands represent within-participant SDs. ***B***, Left, The relationship between intrusion proportion and affect suppression score (No-Think − Baseline) for Negative scenes. Right, Participants who were better at controlling intrusions of unpleasant scenes also showed reduced negative feelings toward them afterward. ★*p* < 0.05. ***C***, Brain areas more engaged by retrieval suppression than by retrieval (No-Think > Think; hot colors) and vice versa (No-Think < Think; cold colors), thresholded at the uncorrected level of *p* < 0.001 for visualization purposes. Pink spheres represent right MFG ROI foci across participants used in subsequent activation, correlation, and DCM analyses (see Materials and Methods). These ROIs were derived from individual local maxima centered around the supramodal control system described by Depue et al. (2015) and are projected onto a common standard space for visualization purposes. Statistical parametric maps were rendered on the top of the PALS human surface using Caret software (Van Essen et al., 2001) (RRID:SCR_006260).

Repeatedly suppressing retrieval of an unwanted memory previously has been shown to decrease its tendency to intrude (Levy et al., 2012; Benoit et al., 2015; Hellerstedt et al., 2016; van Schie and Anderson, 2017). Replicating this finding, we found that participants' control over intrusions improved with practice (Fig. 2*A*). An Emotion × Block ANOVA on participants' intrusion reports for No-Think trials revealed a robust reduction in intrusion proportion over blocks (*F*_(4,92)_ = 11.52, *p* < 0.001). Interestingly, repeated suppressions reduced intrusions comparably for Negative and Neutral scenes (neither the main effect of Emotion, nor its interaction with Block was significant; all *F* values < 2.16), inconsistent with the notion that negative memories are more difficult to control. Indeed, the average intrusion proportions were 0.344 and 0.386 for Negative and Neutral scenes, respectively (corresponding to averages of 28 and 34 intrusions, respectively), indicating that intrusions were numerically less frequent for negative memories. It bears emphasis, however, that these findings were observed in a context in which people were actively trying to suppress memory retrieval; it is possible that Negative memories might be spontaneously retrieved more often when people are not trying to suppress retrieval, a situation that was not studied here. The current findings thus indicate that, when encoding strength is carefully equated (see Procedure), retrieval suppression appears to be comparably effective for materials of Negative and Neutral valence.

We then asked whether participants' ability to gain control over intrusions across blocks was accompanied by persisting suppression of emotional content. To measure whether retrieval suppression influenced later affective responses to scenes, we examined the adjusted SAM valence ratings (Fig. 1*A*; see Materials and Methods). We predicted that suppression would reduce negative valence for Negative scenes, especially for people who were effective at controlling intrusions. This would be reflected in positive affect suppression scores (No-Think − Baseline rating) because higher scores on this measure would indicate that No-Think items had received less negative ratings than had Baseline items. For Neutral scenes, we did not have specific predictions as to whether retrieval suppression should further remove residual negativity (which would actually increase positive feelings toward the Neutral scene), or, instead, simply dampen positive affect for the item.

We first tested whether suppression affected participants' valence ratings for scenes without considering how well participants controlled intrusions. This overall analysis revealed no differences in valence ratings across Think, No-Think, and Baseline conditions for either Negative or Neutral scenes (all relevant comparisons, *p* > 0.1; Table 1). Thus, across all participants, suppression did not consistently affect the perceived valence of the scenes.

**Table 1.** Behavioral data for valence rating and recognition tasks*^a^*

Emotion	Valence rating			Recognition RT (ms)			Recognition accuracy		
	Think	No-Think	Baseline	Think	No-Think	Baseline	Think	No-Think	Baseline
Negative scenes	0.86 (0.35)	0.76 (0.32)	0.78 (0.37)	1444 (338)	1573 (495)	1442 (438)	0.97 (0.10)	0.97 (0.05)	0.98 (0.07)
Neutral scenes	1.14 (0.15)	1.16 (0.17)	1.14 (0.14)	1320 (313)	1356 (295)	1344 (336)	0.97 (0.05)	0.96 (0.06)	0.97 (0.06)

*^a^*Adjusted valence rating and recognition reaction times (RT) and accuracy for Negative and Neutral scenes (SD in parentheses).

Whether suppression affects an image's perceived valence would be expected to depend, however, on how effectively a person mitigates unwanted intrusions through inhibitory control. To test this possibility, we examined the relationship between intrusion frequency and affect suppression scores (No-Think vs Baseline). We first correlated participants' reported intrusion proportion during No-Think trials with their affect suppression scores. We found that participants who were more effective at preventing intrusions of Negative scenes during No-Think trials showed greater affect suppression scores (r-skipped = −0.46, [−0.76, −0.12] bootstrapped 95% CI). We observed no significant relationship between intrusion proportion and affect suppression score for Neutral scenes (r-skipped = −0.09, [−0.35, 0.49] bootstrapped 95% CI).

To further illustrate this relationship, we split participants into two groups according to how well they suppressed intrusions during No-Think trials (Fig. 2*B*). Separating groups in this manner qualitatively illustrates the point made by our significant correlation, that participants differing in putative memory control skill had differing success in reducing negative affect for suppressed scenes. In the high-memory-control group (i.e., low intrusion frequency), we observed significantly reduced negative valence for suppressed Negative scenes compared with Baseline Negative scenes (*t*_(10)_ = 2.29, *p* < 0.05); we observed no evidence that suppression altered the valence of Neutral scenes compared with Baseline Neutral scenes (*t*_(10)_ = 0.3, *p* > 0.1). In striking contrast, in the low memory-control group (higher intrusions), Negative scenes that participants suppressed during No-Think trials were judged as significantly more negative than their Baseline counterparts (*t*_(10)_ = −3.41, *p* < 0.01); again, we found no effect of suppression on affect for Neutral scenes (*t*_(10)_ = −0.4, *p* > 0.1). These relationships between mnemonic awareness and changes in perceived valence were specific to No-Think items: no relationship between mnemonic awareness and valence effects for Think items was observed, regardless of the nature of the scenes (Negative or Neutral).

Although the foregoing analysis suggests that people who are successful at suppressing aversive intrusions also are better at reducing negative affect, an alternative possibility exists. Perhaps the participants who had fewer intrusions in our No-Think task were people who simply found the particular No-Think items they received less upsetting during the encoding phase. If so, low-intrusion participants may, on our final affect measure, show less negative affect for those particular No-Think items than for Baseline items, due to the differing initial affective response to those items, not affect suppression. To scrutinize whether these putative item effects could account for the relationship between intrusion control and affect suppression, we used separate No-Think items to quantify intrusion frequency and affect suppression. First, we randomly split No-Think items into a “sample set” and a “test set.” Second, we computed the intrusion proportion for a given participant using their “sample set” and their affect suppression score using the separate “test set,” rendering these two measurements item-independent. Third, we computed the skipped correlation between these intrusion proportion and affect suppression scores. We repeated this three-step process 1000 times, randomly splitting No-Think items at each iteration to identify sample and test sets. Thus, this procedure yielded a correlation distribution whose reliability could be assessed by computing confidence intervals. If the correlation between intrusion frequency and affect suppression arose simply because less upsetting items will also be less intrusive, then using separate items to quantify these outcomes should eliminate the relationship. This did not occur, however: for Negative scenes, we found a significant relationship between intrusion proportion and affect suppression that was independent of item selection (mean r-skipped = −0.29, [−0.51, −0.03] 95% CI), whereas this relation was absent for Neutral scenes (mean r-skipped = −0.07, [−0.30, 0.20] 95% CI). These findings support the possibility that better intrusion control truly does relate to superior affect suppression (a possibility corroborated by later analyses relating individual differences in brain activity to these scores).

Although the foregoing evidence suggests that suppression disrupts negative affect of No-Think scenes (for people with good memory control), another interpretation exists. Instead of disrupting the negative affective content of No-Think items, suppression may instead increase the positive valence associated with those suppressed items. If so, we would expect a net change in the affect rating for Negative No-Think items toward a more neutral rating, reflecting the new mixture of positive and negative valances of those items. One effective way to rule out this interpretation would be to include positively valenced items in future retrieval suppression studies. If suppression adds positive valence, then positive scenes should be rendered even more positive after suppression; but if suppression disrupts valenced content, suppression should reduce positive affect for suppressed scenes. Although this alternative cannot be ruled out definitively in the current study, data from affective ratings of our Neutral scenes make it is less likely. If suppression added positive valence, we would expect to find the same correlation between our affect suppression score (No-Think − Baseline) and intrusion control for Neutral scenes, but no relationship was found. This finding is more consistent with a disruption of affective content, which is only likely to be present in our negatively valenced scenes. Nevertheless, because we did not include positively valenced scenes in the current design, it is prudent to limit the current conclusions to the disruption of negative affect, not affect more generally.

We also examined how suppression affected performance on the associative recognition test given at the end of the experiment. As expected, accuracy was near to ceiling, given the extensive training of the pairs and the use of a recognition test (accuracy > 95% in all conditions). Nevertheless, recognition reaction times revealed aftereffects of suppressing No-Think pictures. Averaging across valence types, we observed a significant suppression effect for No-Think (mean ± SD, 1464 ± 376 ms) compared with baseline items (1393 ± 367 ms; *t*_(21)_ = 1.89, *p* < 0.05). Considered separately, suppression effects arose for negative scenes (suppression effect, 131 ± 288 ms; *t*_(21)_ = 1.82, *p* < 0.05) but not for Neutral scenes (suppression effect, 12 ± 132 ms; *t*_(21)_ = 0.54, *p* > 0.1), although this interaction did not reach significance (Negative vs Neutral) (*t*_(21)_ = 1.42, *p* = 0.084). These reaction time patterns resemble the recall patterns observed by Depue et al. (2006) who also studied retrieval suppression using the current face-scene pairings (Depue et al., 2006). We also observed a significant main effect of valence on recognition time, with Negative scenes showing slower recognition than Neutral scenes (*t*_(21)_ = 5.06, *p* < 0.001), a pattern observed in prior studies examining recognition of scenes and faces (Keightley et al., 2011).

Overall, the foregoing findings are consistent with the possibility that suppressing Negative scenes reduces their tendency to intrude and may also alter the emotional quality of those memories so that their reappearance triggers less negative affect, at least for people who are proficient in controlling their memories. Interestingly, changes in affect do not arise for Neutral scenes, suggesting that suppressing unpleasant memories may entail additional inhibitory effects not present for Neutral memories. In no case did retrieval during Think trials, by contrast, measurably alter the perceived affect of the scenes. More generally, these findings suggest that inhibitory control might, in parallel, modulate traces in different representational domains (memorial and affective) during efforts to exclude an unwanted memory from awareness. Given this possibility, our goal was then to understand how the right frontoparietal control network contributes to this hypothesized parallel regulation of memory and emotion, and to determine whether these contributions could be related to an inhibitory signal targeted at different substructures within the MTL.

### Fronto-MTL regions are engaged during the suppression of intrusive memories

Before addressing whether retrieval suppression modulates regions related to both memory and affect, we confirmed the engagement of the right frontoparietal control network and the disengagement of MTL during retrieval suppression. First, we contrasted No-Think and Think trials aggregating conditions over both Negative and Neutral scenes (*P*_FWE_ < 0.05; Fig. 2*C*). Consistent with previous findings, we observed more activation during No-Think trials in a large right-lateralized network, including the MFG, dorsal portion of the anterior cingulate cortex, superior frontal medial gyrus, inferior frontal gyrus, insula, superior frontal gyrus, and inferior parietal cortex. Critically, a right dorsolateral prefrontal cortex cluster, centered on the anterior MFG and previously noted for its involvement in direct memory suppression, survived whole-brain voxelwise correction (*x* = 30, *y* = 48, *z* = 16; *z*_max_ = 6.15; 1591 voxels; *P*_FWE_ < 0.001; for complete whole-brain analyses, see Table 2). Thus, in agreement with previous findings using other types of suppressed material (e.g., words, objects, faces, places), the right MFG was engaged in the suppression of both Negative and Neutral scenes (Depue et al., 2007; Benoit et al., 2015). We observed no interaction surviving whole-brain correction between memory control (i.e., No-Think > Think) and Emotion, nor did we observe differences between Negative and Neutral scenes in the No-Think condition. These results suggest that a common control network suppresses retrieval regardless of the valence of the unwanted memory.

**Table 2.** Regions showing a difference in activity between Think and No-Think trials*^a^*

Anatomical description	No. of voxels	MNI coordinates (mm)			*Z*	*P*_FWE_
		*x*	*y*	*z*		
No-Think > Think						
Right inferior frontal gyrus	1859	48	20	−2	Inf	<0.001
Right superior frontal gyrus	2011	14	14	58	7.48	<0.001
Dorsal portion of the anterior cingulate cortex		4	24	32	6.88	<0.001
Right inferior parietal cortex	662	60	−46	32	7.28	<0.001
Right middle frontal gyrus	1591	42	24	36	6.34	<0.001
Right middle frontal gyrus (anterior)		30	48	16	6.15	<0.001
Right middle frontal gyrus (anterior)		28	48	32	5.86	<0.001
Right middle frontal gyrus (posterior)		44	14	41	5.74	<0.001
Right precentral gyrus		50	2	40	5.29	<0.005
Left insula	753	−46	16	0	7.04	<0.001
Left inferior frontal gyrus		−56	20	2	5.96	<0.001
Left middle frontal gyrus	23	−28	52	14	4.93	<0.01
Right superior temporal gyrus	21	54	−28	10	4.74	<0.05
Left inferior parietal cortex	6	−52	−58	44	4.70	<0.05
Right striatum	18	16	2	8	4.64	<0.05
Think > No-Think						
Left cuneus	4387	0	−84	24	7.47	<0.001
Left middle occipital gyrus	209	−38	−78	32	6.31	<0.001
Left postcentral gyrus	19	−40	−24	62	4.70	<0.05
Right fusiform gyrus	28	32	−36	−16	5.04	<0.005
Left fusiform gyrus	19	−30	−38	−14	4.65	<0.05

*^a^*Peak coordinates of the regions showing greater activity for No-Think relative to Think items (and vice versa) across Negative and Neutral scenes at *P*_FWE_ < 0.05 (whole-brain).

We then examined how retrieval suppression affected activity in the right MFG ROI (see ROI selection). We focused our analysis on No-Think trials, dividing them into Intrusions and Non-Intrusions, based on what participants reported on the intrusion scale immediately following each trial. We note the contrast used to select this ROI (collapsing across all No-Think trials vs Think trials) is orthogonal to the comparison of Intrusion versus Non-Intrusion trials (upregulation effect), thus avoiding circularity issues when we compared those conditions. A two-factor ANOVA with Emotion (negative, neutral) and Awareness (Intrusions vs Non-Intrusions) as factors revealed neither a main effect of Awareness nor the presence of a significant interaction between the two factors (all *F* values <0.94).

**Figure 3.**
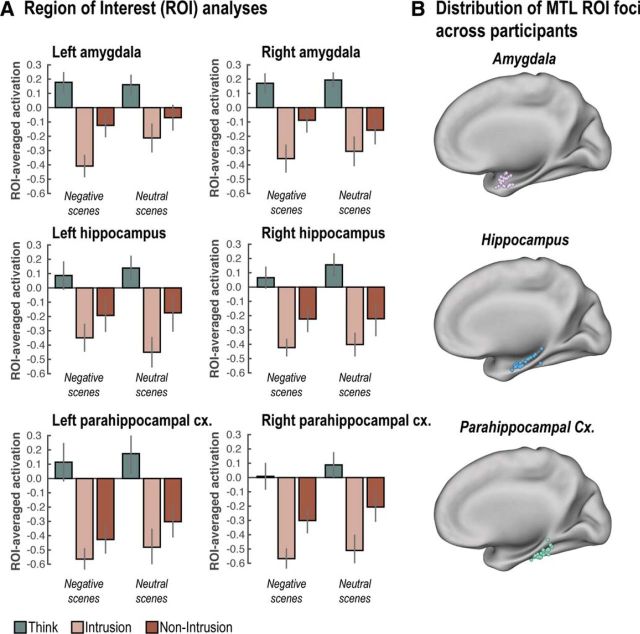
MTL downregulation. ***A***, Suppressing scene memories reduced activity across the whole MTL overall. Additionally, we observed more pronounced downregulation in these MTL regions during suppression attempts that were accompanied by intrusions. ***B***, Distribution of MTL ROI foci across participants once projected back to MNI space. Error bars indicate SEM.

We then examined how retrieval suppression affected activity in MTL ROIs. Previous studies have found more pronounced downregulation of hippocampal activity during retrieval suppression when memories involuntarily intrude into consciousness compared with when they do not (Levy and Anderson, 2012; Benoit et al., 2015). We next sought to replicate these findings to determine whether they generalize to the amygdala, and to examine whether they interact with valence. In agreement with prior findings, a Hemisphere × Region × Emotion × Awareness (Intrusion vs Non-Intrusion) ANOVA showed a significant decrease in activity during Intrusion relative to Non-Intrusion trials (*F*_(1,21)_ = 8.79, *p* < 0.01). This downregulation of activity during intrusions varied with both Region and Emotion, as revealed by a significant three-way interaction of Awareness with Region and Emotion (*F*_(1,42)_ = 3.25, *p* < 0.05). This interaction was driven mainly by two effects (Fig. 3): (1) in both hemispheres, the amygdala showed greater downregulation for negative than for neutral scenes; and (2) the left hippocampus and right parahippocampus showed greater downregulation than the other regions.

Together with engagement of the right frontoparietal network, these findings support the possibility that retrieval suppression recruited the putative memory inhibition network to suppress episodic retrieval of intrusive memories. Critically, they further show that previous demonstrations of intrusion-dependent downregulation (Levy and Anderson, 2012) also generalize to the amygdala when aversive scenes are suppressed. This clear evidence for modulation of the amygdala (together with clear negative valence ratings for Negative items, mean = 2.2, on our final valence rating test), suggests that our training regimen, involving multiple presentations and tests of our face-scene pairs, did not eliminate the affective properties of our negative scenes or the need to suppress emotional responses to them. Importantly, this joint modulation of the amygdala and hippocampus in response to intrusive memories provides strong preliminary support for the parallel regulation of emotion and memory by retrieval suppression. We explore this parallel regulation hypothesis in greater depth next.

### Intrusion regulation and affect suppression score depend on common prefrontal regions and MTL downregulation

**Figure 4.**
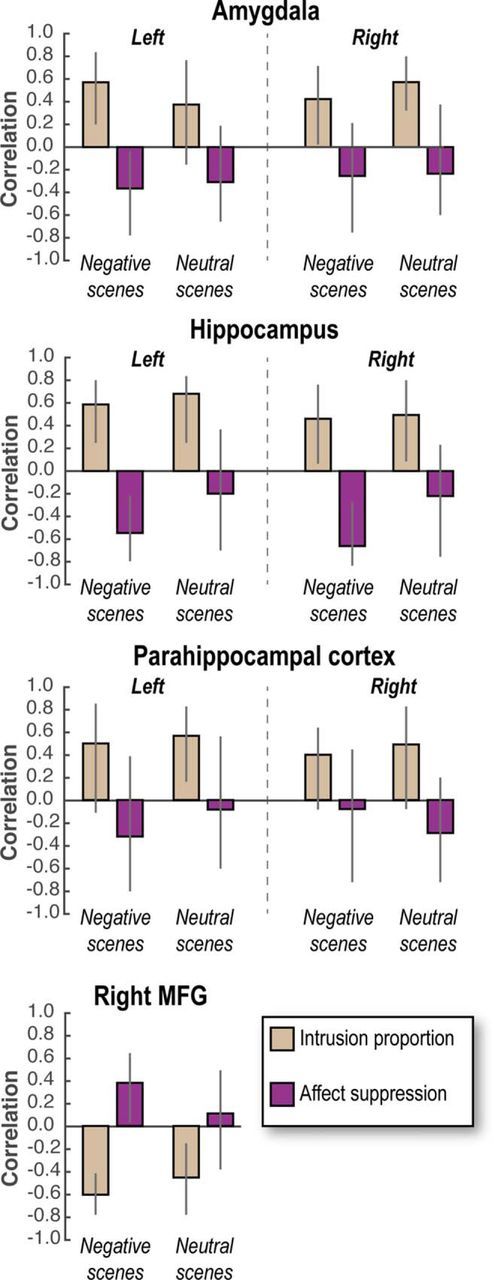
Brain/behavior correlations. Pearson correlation (skipping bivariate outliers) between affect suppression/intrusion proportion and neural marker of memory suppression (Intrusion − Non-Intrusion) for each scene type. For neural markers of memory suppression in the MTL, negative (lower) scores are assumed to indicate more successful suppression of activity (Intrusion − Non-Intrusion is a downregulation in most cases, suggesting control); in contrast, neural markers for memory suppression in MFG are upregulations of activity (again, Intrusion − Non-Intrusion), and so positive scores are assumed to indicate greater engagement of control. Higher behavioral scores for affect suppression scores indicate greater reduction in negative valence for No-Think compared with Baseline items (i.e., No-Think − Baseline); in contrast, higher intrusion scores indicate worse control over intrusions. Together, these considerations indicate that, in MTL, positive correlations for intrusions signify that downregulations predict fewer intrusions, whereas negative correlations for affect suppression signify that downregulations predict reduced affect. In MFG, the direction of correlations is expected to invert because greater control is indicated by upregulation. Error bars indicate 99.3% bootstrapped CI corrected for multiple comparisons across ROIs. Significant correlations occur when the CI does not encompass zero.

We next examined whether people's ability to suppress intrusive memories and alter their perceived valence (i.e., affect suppression score) might depend on a common right frontoparietal network that modulates MTL activity. We first used the standard brain-behavior correlation method using a robust statistical approach as described by Pernet et al. (2013) (see Materials and Methods). The downregulation and upregulation effects (Intrusion vs Non-Intrusion) were extracted in MTL and right MFG ROIs, respectively. We correlated these neural markers of memory suppression with the behavioral markers of affect regulation and intrusion control. Figure 4 reports in detail the outcome of these correlations after correcting for multiple comparisons. We focus on the skipped correlation, which accurately controls for outliers. Broadly, during the suppression of intrusive scenes, both downregulated activation in the hippocampus and amygdala and upregulation in right MFG were associated with reduced intrusion frequency for both Negative and Neutral scenes and increased affect suppression scores only for Negative scenes.

The foregoing ROI-behavior correlation analyses provide initial support for a shared control mechanism underlying mnemonic and affective regulation. However, this approach does not allow us to ascertain whether the exact same voxels contribute to regulation in both domains. Indeed, it may be that different sets of voxels across the MTL or the control network produce relationships for affect and intrusion suppression. We therefore used the behavioral PLS correlation method, which identifies sets of voxels (i.e., dimensions) that express quantitatively different relationships with behavioral measures (see Materials and Methods). Behavioral PLS takes the behavioral measurements (here, our intrusion frequency and affect suppression measures) together with fMRI activations observed at each voxel (based here on the Intrusion vs Non-Intrusion contrast, our neural marker of intrusion control), and calculates a correlation matrix, across participants. Singular value decomposition is then applied to this correlation matrix to produce orthogonal LVs that optimally represent the relationship between intrusion control activations and behavioral markers of mnemonic and affective control. These LVs are extracted in the order of the amount of covariance they explain between intrusion control activations and behavioral measures. On one hand, if different sets of voxels express a distinct and significant relationships with affect suppression scores and intrusion proportions, respectively, this technique would identify two significantly separate LVs. On the other hand, if a common set of voxels express a common relationship with both affect suppression score and intrusion proportion, a unique LV would emerge from this analysis.

Because the outcome of the PLS analysis might vary with the valence of our materials, we conducted separate analyses for Negative and Neutral scenes. For Negative scenes, the first LV accounted for 76% and 79% of the covariance between intrusion control activations and behavioral measures of intrusion proportion and affect suppression within the frontoparietal retrieval suppression network and the MTL mask, respectively (*p* < 0.05). The first LV was significantly different from random noise as assessed by permutation testing (*p* < 0.05). For Neutral scenes, the first LV accounted for 73% and 68% of the covariance between behavioral measurements and the prefrontal retrieval suppression network and the MTL mask, respectively (*p* < 0.05). A second LV was not significant for either Negative or Neutral scenes.

The existence of a significant LV that accounts for a large percentage of the covariance between intrusion-control activations and both of our behavioral measures indicates that some voxels within the prefrontal cortex and MTL are related to mnemonic and/or affective control of unpleasant memories. They do not, however, specify how brain activation relates to those measures. To allow for more clear interpretation of the role of these voxels, we next computed “voxel salience” (the voxels that most contributed to our key LV) and a brain score for each participant (see Materials and Methods). A brain score indicates how much a given participant expresses the multivariate spatial pattern of correlation between intrusion control activations and behavioral measures of mnemonic and affective control captured by an LV. Thus, correlations between brain scores and behavioral measurements help to identify the direction and the strength of the relationship captured by a given LV (and thus the corresponding voxel salience over that LV).

**Figure 5.**
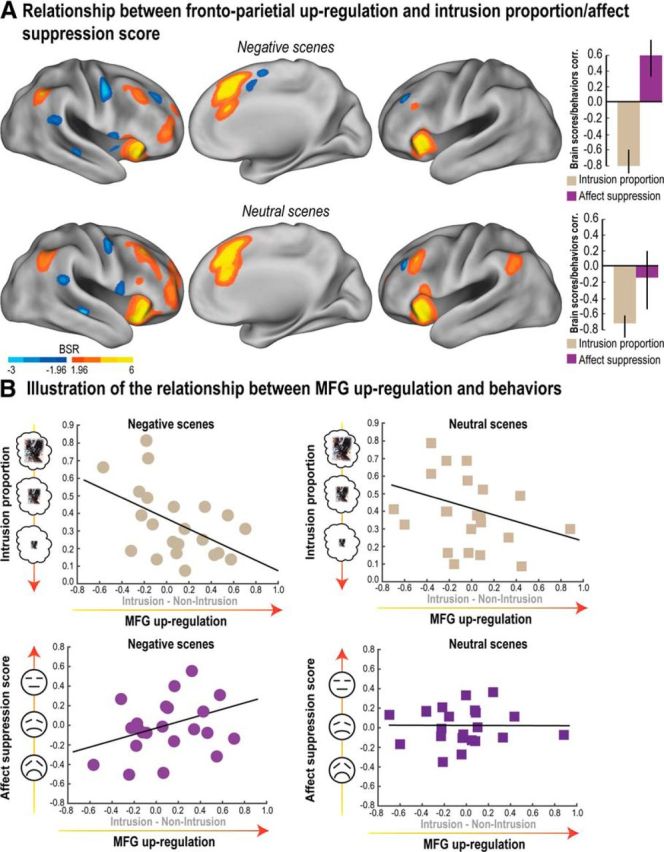
Relationship between neural markers of inhibitory control and the reduction of mnemonic awareness/affective response. Outcome of the PLS analysis for both Negative and Neutral scenes (conducted within the retrieval suppression network; see Materials and Methods) between intrusion-related upregulation (Intrusion − Non-Intrusion) and behavioral measures (intrusion proportion and affect suppression score). ***A***, Voxels showing a significant pattern of brain/behavior correlations as revealed by the first (significant) LV were identified using a BSR threshold higher/lower than 1.96/−1.96, respectively (i.e., *p* < 0.05). Correlations between participants' brain scores and behavioral measures for the first significant LV are also reported in ***A***. Error bars indicate bootstrapped 95% CI. Brain scores reflect the contribution of each participant to a given LV. The correlation between brain scores and behaviors thus reveals the meaning of the LV. ***B***, Scatter plots observed in the right MFG illustrating the relationship captured by PLS analysis between the upregulation (Intrusion − Non-Intrusion) and behavioral scores for Negative and Neutral scenes. These findings reveal voxels whose upregulation is associated with reduced intrusion frequency for both Negative and Neutral scenes and also with increased affect suppression score only in the case of Negative scenes (reduced negative affect for suppressed images). BSR maps were rendered on the top of the PALS human surface using Caret software (Van Essen et al., 2001) (RRID:SCR_006260).

Critically, within the frontoparietal control network, we found that, for negative scenes, participants' brain scores for the first LV correlated positively with their affect suppression scores (*r* = 0.60 *p* < 0.05, [0.35, 0.79] bootstrapped 95% CI) and negatively with intrusion proportion (*r* = −0.79, *p* < 0.05, [−0.91, −0.60] bootstrapped 95% CI; Fig. 5*A*). Importantly, this finding indicates that for those voxels having a positive salience, upregulation during Intrusions (vs Non-intrusions) negatively correlated with intrusion frequency (i.e., better mnemonic suppression), and additionally positively correlated with affect suppression score (i.e., better affective suppression) for Negative scenes (for an illustration of this pattern, see Fig. 5*B*). These patterns are inverted for voxels associated with a negative salience. Voxels within our retrieval suppression mask associated with a significantly positive salience (using a bootstrapping procedure; see Materials and Methods) were localized across the entire control network, including left and right MFG, dorsal portion of the anterior cingulate cortex, superior frontal medial gyrus, inferior frontal gyrus, insula, and inferior parietal cortex (Fig. 5*A*; Table 3). For Neutral scenes, participants' brain scores for the first LV also correlated negatively with intrusion proportion (*r* = −0.72, *p* < 0.05, [−0.87, −0.54] bootstrapped 95% CI) but, in contrast to findings with negative scenes, showed no significant correlation with their affect suppression scores (*r* = −0.16, [−0.54, 0.19] bootstrapped 95% CI; Fig. 5*A*).

**Table 3.** Control network regions showing a significant pattern of brain/behavior correlations as revealed by the first latent variable of the PLS analysis*^a^*

Anatomical description	No. of voxels	MNI coordinates (mm)			BSR	*p* value	R-Intrusion proportion	R-Affect suppression
		*x*	*y*	*z*				
Negative scenes								
Left superior frontal medial gyrus	1865	2	24	44	9.42	0.0000000	−0.7822	0.5294
Right insula/inferior frontal gyrus	1072	28	24	0	6.95	0.0000000	−0.6010	0.5700
Left insula/inferior frontal gyrus	520	−32	18	−2	5.40	0.0000000	−0.5193	0.4027
Right inferior parietal cortex	182	52	−54	46	4.82	0.0000007	−0.6991	0.3424
Right middle frontal gyrus (anterior)	215	38	50	8	4.67	0.0000015	−0.5491	0.4638
Right middle frontal gyrus (posterior)	143	40	24	42	4.08	0.0000228	−0.5648	0.2996
Left middle frontal gyrus	10	−34	50	4	2.99	0.0013989	−0.4767	0.2461
Right superior frontal gyrus	29	22	56	24	2.40	0.0082399	−0.3670	0.4384
Right middle frontal gyrus	67	32	36	46	−4.27	0.0000099	0.4337	−0.4310
Right precentral gyrus	108	50	−4	50	−4.16	0.0000157	0.6450	−0.5005
Right rolandic operculum	52	56	6	2	−3.59	0.0001665	0.4331	−0.4357
Right supplemental motor area	27	8	−2	58	−3.12	0.0009183	0.4253	−0.4943
Right superior temporal gyrus	67	54	−40	22	−3.06	0.0010909	0.3099	−0.4957
Right middle temporal gyrus	23	48	−22	−8	−2.52	0.0059107	0.2889	−0.4060
Neutral scenes								
Right dorsal portion of the anterior cingulate cortex	2465	6	28	34	10.2	0.0000000	−0.6823	−0.2088
Right insula/inferior frontal gyrus	1099	34	20	−14	10.1	0.0000000	−0.6789	−0.1806
Right middle frontal gyrus (posterior)	444	38	24	36	7.39	0.0000000	−0.5318	−0.1780
Left insula/inferior frontal gyrus	906	−30	18	−16	5.80	0.0000000	−0.5960	−0.0471
Right middle frontal gyrus (anterior)	611	40	54	4	5.77	0.0000000	−0.5453	−0.2580
Right angular gyrus	335	48	−58	40	4.59	0.0000023	−0.6438	−0.0113
Left middle frontal gyrus	129	−38	26	34	3.95	0.0000393	−0.4905	−0.2614
Right superior frontal gyrus	17	18	56	−8	3.38	0.0003675	−0.4326	0.0491
Right superior frontal gyrus	17	18	6	56	3.11	0.0009341	−0.2907	−0.1668
Left supramarginal gyrus	131	−60	−54	30	3.03	0.0012034	−0.4391	0.1107
Right striatum	23	16	12	0	2.86	0.0020956	−0.3100	−0.1602
Left superior frontal gyrus	11	−26	54	16	2.57	0.0050181	−0.3600	0.0185
Right middle temporal gyrus	13	60	−32	−8	2.47	0.0066930	−0.4098	0.0604
Right precentral gyrus	44	46	−6	48	−2.85	0.0021916	0.5611	−0.2745
Right superior temporal gyrus	20	50	−26	−4	−2.47	0.0067875	0.4536	−0.2466
Right supramarginal gyrus	13	56	−36	30	−2.39	0.0083475	0.4167	−0.1571
Right superior temporal gyrus	17	56	−44	18	−2.31	0.0105426	0.4501	−0.2108

*^a^*Peak coordinates of the control regions significantly loading on the first LV. Rightmost columns indicate the correlation between cluster upregulation (Intrusion vs Non-Intrusion) and intrusion proportion/affect suppression score.

**Figure 6.**
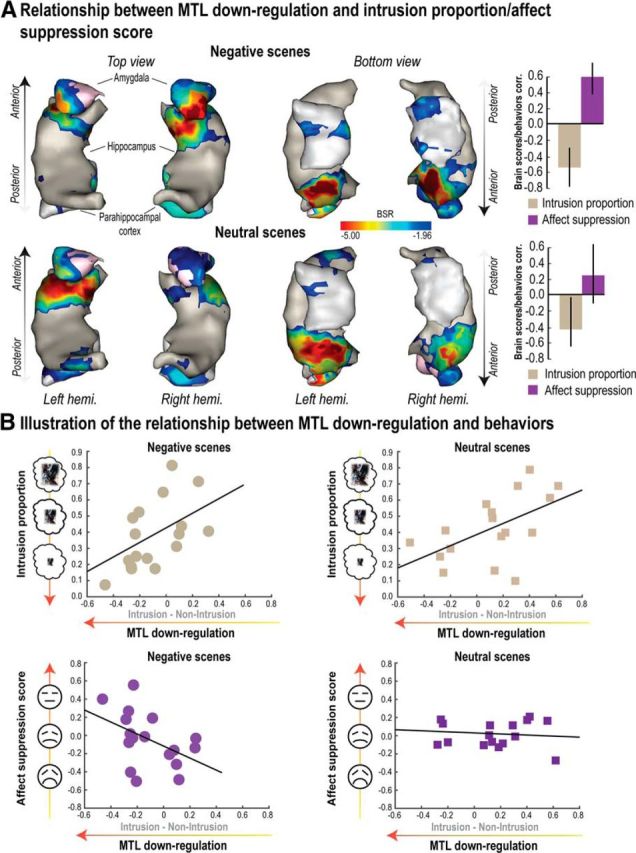
Relationship between MTL downregulation and reductions of mnemonic awareness/affective response. Outcome of the behavioral PLS analysis for both Negative and Neutral scenes conducted within the MTL, including bilateral amygdala, hippocampus, and parahippocampal cortex. PLS correlations were tested with the MTL contrast map for the differences between Intrusion and Non-Intrusion conditions on one hand (i.e., downregulation), and behavioral scores (intrusion proportion and affect suppression, i.e., the reduction of negative feelings for No-Think items relative to Baseline) on the other hand. ***A***, Voxels significantly associated to the first significant LV and whose downregulation significantly correlated with intrusion proportion for both Negative and Neutral scenes, as well as with affect suppression exclusively for Negative scenes. Error bars indicate bootstrapped 95% CI. Voxels were identified using a BSR higher or lower than 1.96/−1.96, respectively (i.e., *p* < 0.05). Correlations between participants' brain scores and behaviors for the first significant LV are also reported in ***A***. Clusters of BSR exceeding threshold were rendered onto a 3D reconstruction of a standard MTL template. The 3D representation of the MTL was obtained by transforming MTL binary masks into 3D meshes using “Anatomist/BrainVISA” software (http://www.brainvisa.info/; RRID:SCR_007354). ***B***, Scatter plots illustrating the relationship captured by PLS analysis between the downregulation observed in the MTL cluster (***A***) and behavioral scores for both Negative and Neutral scenes.

Within the MTL mask, we found a similar pattern for Negative scenes, participants' brain scores for the first LV correlated positively with affect suppression score (*r* = 0.59, *p* < 0.05, [0.38, 0.79] bootstrapped 95% CI) and negatively with intrusion proportion (*r* = −0.55, *p* < 0.05, [−0.78, −0.28] bootstrapped 95% CI), whereas for Neutral scenes, brain scores only correlated with intrusion proportion (*r* = −0. 40, *p* < 0.05, [−0.66, −0.02] bootstrapped 95% CI) but not with affect suppression (*r* = 0.24, [−0.12, 0.64] bootstrapped 95% CI; Fig. 6*A*). Given that most voxels had a reliably negative salience within the MTL mask (located mainly in the bilateral anterior hippocampus and amygdala; Fig. 6*A*, Table 4), the foregoing pattern means that, during the suppression of intrusive scenes, downregulating activation in these voxels was associated with decreased intrusion frequency for Negative and Neutral scenes and greater affect suppression scores (but only for Negative scenes; for an illustration of this pattern, see Fig. 6*B*). No voxels within the MTL were associated with a significant positive salience (i.e., the opposite pattern).

**Table 4.** MTL regions showing a significant pattern of brain/behavior correlations as revealed by first latent variable of PLS analysis*^a^*

Anatomical description	No. of voxels	MNI coordinates (mm)			BSR	*p* value	R-Intrusion proportion	R-Affect suppression
		*x*	*y*	*z*				
Negative scenes								
Right hippocampus/amygdala	580	24	−12	−18	−7.04	0.0000000	0.5268	−0.5867
Left hippocampus/amygdala	336	−30	−8	−16	−6.18	0.0000000	0.5113	−0.5489
Right parahippocampal cortex	202	20	−36	−14	−4.25	0.0000109	0.4385	−0.6719
Left parahippocampal cortex	178	−20	−32	−14	−4.19	0.0000141	0.6096	−0.4909
Neutral scenes								
Left amygdala/hippocampus	487	−30	0	−22	−6.89	0.0000000	0.3916	−0.2846
Right hippocampus/amygdala	499	34	−18	−8	−6.63	0.0000000	0.3566	−0.2604
Right parahippocampal cortex	97	28	−38	−12	−4.67	0.0000015	0.3007	−0.3681
Left hippocampus/parahipp. cortex	173	−20	−34	0	−4.16	0.0000162	0.4173	−0.2385

*^a^*Peak coordinates of the MTL regions significantly loading on the first LV. Rightmost columns indicate the correlation between cluster downregulation (Intrusion vs Non-Intrusion) and intrusion proportion/affect suppression score.

Together, the foregoing results strongly support the hypothesis that retrieval suppression does not simply reduce the accessibility of episodic memories but can also alter negative affect. In particular, increased activation in the right lateral PFC region previously associated with retrieval suppression predicted reduced intrusion frequency as well as increased valence suppression for those suppressed images. Thus, individuals who adaptively upregulated right MFG in response to intrusions could purge the unwanted trace from awareness and disrupt emotional content of suppressed traces at the same time, decreasing the likelihood of those traces reentering consciousness and triggering upsetting thoughts. Notably, this evidence for colocalization of mnemonic and affective regulation is unlikely to be an artifact of poor spatial resolution masking what might be two similarly located but distinct forms of control. Because we related activations during retrieval suppression to mnemonic and affective measures on a voxel-by-voxel basis, these findings likely indicate the operation of a shared mechanism. These findings add force to the results of previous within-subjects conjunction analyses performed on separate affect regulation and retrieval suppression tasks (Depue et al., 2016) and specifically link activations in right MFG to intrusion control and affect regulation of negative memories.

Critically, our findings further reveal that the ability to control intrusive memories and reduce the negative affect associated with them is related to downregulation of a shared set of voxels within the MTL. These shared modulatory targets were localized to anterior hippocampus and amygdala. There is evidence for greater anatomical and functional connectivity between anterior hippocampus and amygdala (Poppenk et al., 2013), relative to mid or posterior hippocampus supporting this type of interaction. One interpretation of our findings, therefore, is that cue-driven reinstatement of aversive scenes in the hippocampus may have triggered affect-related activity in the amygdala, prompting the targeting of that structure for inhibitory control.

### Modulation of MTL activity is parallel and reactive

Although the current findings suggest that suppressing intrusive memories disrupts episodic and emotional traces, it does not establish specifically that right MFG modulates MTL activity, nor whether such modulation acts in parallel on both memory and emotion-related sites. It is possible, for example, that MTL regions were simply not engaged during intrusions, rather than being suppressed; participants might have shifted attention away from the reminder cue if they sensed that they could not control a memory. Such perceptual avoidance might limit sensory input into the MTL, reducing activity during intrusions relative to Non-Intrusions. Moreover, even if right MFG modulates MTL activity, we do not know which structures are affected. On the one hand, reduced amygdala activation during intrusions might reflect reduced afferent input from memory-related regions (i.e., hippocampus and/or parahippocampal cortex), caused by suppressing the latter regions. By this memory-regulation account, reduced amygdala is the downstream effect of inhibitory modulation elsewhere. On the other hand, modulation instead may target the amygdala and be echoed in memory-related regions. Our parallel regulation hypothesis, however, predicts that neither mnemonic nor affective regulation alone accounts for the neural data; rather, it states that a top-down mechanism regulates emotion-related processing in the amygdala in addition to regulating retrieval processes supported by the hippocampal and parahippocampal cortices. Such parallel regulation should be particularly pronounced when memories intrude into awareness and control must be engaged reactively to counter unwanted awareness.

We first used DCM and BMS to determine whether the right MFG modulated MTL activity during No-Think trials and, if so, which structures it affected: the right (ipsilateral) amygdala, hippocampus, and/or parahippocampal cortex (see Materials and Methods). We chose DCM as a method in part because any putative causal influence that MFG might exert on activity in the hippocampus and amygdala would necessarily be mediated polysynaptically (e.g., perhaps via structures such as the anterior cingulate cortex) (Anderson et al., 2016). Because the precise anatomical pathway mediating this top-down influence has not yet been identified, and because DCM can evaluate effective connectivity without presupposing direct anatomical connections between two structures, this method is well suited to test our mechanistic hypotheses: hypotheses that mainly concern whether retrieval suppression engages MFG to achieve both mnemonic and affective regulation in parallel.

**Figure 7.**
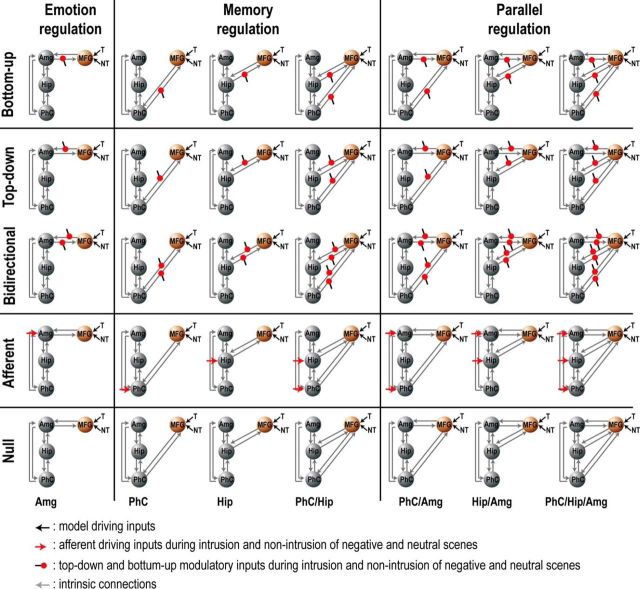
DCM model space. DCM models were organized into two families. The first (the Modulatory family) divided the model space into five subgroups differing according to whether the intrinsic connection from the right MFG was modulated or not by No-Think trials (modeled as 3 s short epochs separately for Intrusion and Non-Intrusion of each emotion type). Subgroups 1–5 of this family (rows 1–5) either included modulation on bottom up (e.g., hippocampus to MFG, row 1), top-down (row 2), or bidirectional (row 3) connections; or no modulation, but variable afferent input to MTL regions from a source independent of MFG (row 4); or no modulation at all (row 5). The second family (the Regulation family) divided the model space into families according to modulatory targets. Subgroups 1–3 of this family (columns 1–3) include the Emotion Regulation family (left, amygdala modulation only), the Memory Regulation family (middle, modulation of hippocampus, parahippocampal cortex, or both), and the Parallel regulation family (right, modulation of amygdala, and other memory-related regions). After estimating all 35 models for each participant, we performed the group BMS as implemented in SPM12 (version DCM12 revision 4750; RRID:SCR_007037). This produces the exceedance probability (i.e., the extent to which each model is more likely than any other considered model) and expected posterior probability (i.e., the probability of a model generating the observed data). By positing connectivity relationships between MFG and these MTL structures in our DCM models, we do not presuppose direct anatomical connections (an assumption that DCM's analytical method does not require); rather, we are modeling the data to evaluate the existence of a (potentially) polysynaptic and directional causal influence of each region on the activity of others to which it is connected.

To evaluate which regions are modulated during suppression, we compared models lacking any modulation (i.e., “null” models), to those characterized by top-down (MFG → MTL), bottom-up (MTL → MFG), bidirectional (i.e., top-down and bottom-up), or afferent sources of modulation (Fig. 7). We intended our “afferent” models to capture the possibility that MTL activation differences simply reflected variations in bottom-up sensory input, without modulation of connectivity between MFG and MTL. Top-down or bottom-up modulation models hypothesized that activation differences across Intrusion and Non-Intrusion conditions were driven by modulatory influences on MFG → MTL or MTL → MFG connections, respectively. The findings were unambiguous: BMS overwhelmingly favored models including top-down modulation, with an exceedance probability (EP) = 0.999 and expected posterior probability (EPP) = 0.80; in contrast, “null,” “bottom-up,” and “afferent” models had a EP and a EPP of 0.0/0.0/0.001, and 0.039/0.039/0.12, respectively. Exceedance probability refers to the extent to which a model is more likely in relation to other models considered, whereas expected posterior probability is the probability of a model generating the observed data.

Having evidence for modulation, we then used BMS to compare the 14 remaining models within the “top-down” subfamily (including top-down and bidirectional models). We divided these into subfamilies to distinguish among our three alternatives: the emotion regulation family (models with modulation of the amygdala), the memory regulation family (models with modulation of hippocampus, the parahippocampal cortex, or both), and the parallel regulation family (models with modulation of the amygdala and the hippocampus, the amygdala, and the parahippocampal cortex, or all three structures; Fig. 7). Critically, in comparing these three families, BMS provided strong evidence favoring parallel regulation models (EP = 0.99, and EPP = 0.63) over either emotion regulation (EP = 0.003, and EPP = 0.16) or memory regulation models (EP = 0.009, and EPP = 0.20). These data thus strongly support our key hypothesis that retrieval suppression drives modulatory signals from the right MFG to MTL regions (together with a bottom-up influence), and that suppression not only affects episodic retrieval processes, but also affective processes.

We also sought to determine whether the greater downregulation of BOLD signal in the MTL during intrusions reflected stronger action of inhibitory control. To evaluate this hypothesis, we extracted DCM coupling parameters and applied Bayesian model averaging on the preferred Parallel family. From this model family, we extracted 12 parameters for each of our participants, that quantified the modulatory influence of MFG on the different target regions (i.e., PhC/Hip/Amg), for materials differing in valence (i.e., Negative/Neutral), during differing levels of intrusiveness (i.e., Intrusion/Non-Intrusion) that we measured in our design. We first tested whether these parameters differed significantly from zero using 5000 bootstrapping resamplings of the sum of intrinsic connections and modulatory parameters (i.e., DCM.A + DCM.B), and applying Bonferroni correction across the 12 parameters (leading to 99.6% CI). One participant with aberrant coupling parameters deviating >4 SDs of the mean was excluded from this analysis. For negative scenes, memory intrusions were associated with significant negative coupling to the parahippocampal cortex ([−0.97, −0.17] bootstrapped 99.6% CI), to the hippocampus ([−0.68, −0.04] bootstrapped 99.6% CI), and to the amygdala ([−0.87, −0.08] bootstrapped 99.6% CI; Table 5). For intrusions of Neutral scenes, coupling parameters to the parahippocampal cortex ([−1.06, −0.14] bootstrapped 99.6% CI), and to the hippocampus ([−0.70, −0.05] bootstrapped 99.6% CI) did also differ significantly from zero, but not for the amygdala ([−0.70, 0.51] bootstrapped 99.6% CI; Table 5). Modulatory parameters never differed reliably from zero during Non-Intrusion trials regardless of valence. These findings suggest that negative coupling between the MFG and MTL regions was generally greater during Intrusions than Non-Intrusions, and spanned both memory and emotion-related regions for negative materials.

**Table 5.** DCM intrinsic and modulatory parameters*^a^*

	DCM.A Intrinsic	DCM.A + DCM.B			
		Negative		Neutral	
		I	NI	I	NI
MFG → Phc	−0.22 (0.52)	−0.55* (0.69)	−0.36 (0.81)	−0.49* (0.76)	−0.14 (0.54)
MFG → Hip	−0.16 (0.35)	−0.32* (0.54)	−0.25 (0.62)	−0.32* (0.52)	−0.19 (0.48)
MFG → Amg	−0.09 (0.29)	−0.47* (0.63)	−0.13 (0.60)	−0.18 (0.97)	−0.39 (0.61)

*^a^*Mean DCM.A (intrinsic) and effective connectivity (DCM.B + DCM.A) between MFG and MTL regions for Intrusions (I) and Non-Intrusions (NI) by scene valence (SD in parentheses).

*Significant coupling parameters (99.6% bootstrapped CI corrected for multiple comparisons using Bonferroni correction).

To verify the impression that top-down coupling parameters differed between Intrusion and Non-Intrusion trials, we computed a Region (PhC/Hip/Amg) × Emotion (Negative/Neutral) × Awareness (Intrusion/Non-Intrusion) ANOVA. As predicted, we observed a significant main effect of Awareness on coupling parameters which were, on average, more negative during Intrusions (mean ± SD, −0.39 ± 0.38) than during Non-Intrusions (−0.24 ± 0.45) (*F*_(1,20)_ = 3.82, *p* < 0.05). No further main effects or interactions were significant (all *F* values < 0.92), except for the Region × Emotion × Awareness interaction, which approached significance (*F*_(1,20)_ = 2.87, *p* = 0.068). This latter trend in part reflects greater negative coupling to the amygdala during intrusions of negative, compared with neutral scenes (Table 5).

Together, the results of our BMS and Bayesian model averaging analyses indicate the existence of modulatory influences of MFG on MTL structures and that such modulation is inhibitory in nature. During the suppression of negative memories, this modulation not only affects regions critical to episodic memory, but also the amygdala, and is particularly pronounced when memories intrude into awareness and need to be purged. These findings support the parallel regulation of memory and emotion by inhibitory control mechanisms that are reactive in nature, suppressing awareness of intrusive memories.

## Discussion

When unpleasant memories intrude into awareness, people often suppress their retrieval to regulate their emotional state. Although considerable work has addressed the mechanisms of retrieval suppression, this work has not examined how suppression alters people's emotional state. Does suppression only target episodic memories, disrupting mnemonic awareness, reducing input to mechanisms that would have driven unpleasant emotions? Or does it inhibit both episodic memories and affective traces? The present data support the latter view: suppressing unpleasant remindings not only disrupts memories supported by the hippocampus and parahippocampus, but also emotional traces that depend on the amygdala, and these parallel effects arise from a shared inhibitory mechanism mediated by the right dorsolateral prefrontal cortex.

### Parallel regulation of memory and affect

Several key findings point to the parallel regulation of memory and affect. First, behavioral and neural data suggest that suppression reduced affective responses to unpleasant memories. Behaviorally, participants who controlled intrusions well showed greater reductions in negative affect for the suppressed unpleasant scenes. This suggests that suppressing episodic retrieval engages a mechanism that also affects emotional traces. Supporting this interpretation, a PLS analysis revealed common areas within the right dorsolateral and ventrolateral prefrontal cortex that predicted both how well participants reduced intrusions and negative affect for suppressed scenes. Intrusions increased activation in these regions, suggesting a colocalized control mechanism that suppresses both types of content. Critically, PLS analyses also identified regions within the anterior hippocampus and amygdala that predicted both intrusion control and affect regulation. Unlike in the prefrontal cortex, voxels in these regions were downregulated, suggesting that inhibitory mechanisms suppressed their activity. Together, these behavioral and neural findings suggest that suppression mechanisms triggered by intrusions not only disrupt episodic memories (Levy and Anderson, 2012) but also blunt negative feelings about images in an enduring way, by a shared mechanism supported by lateral prefrontal cortex.

Of course, suppression-related activations do not necessarily imply that the prefrontal cortex reduced MTL activation. Reduced activations may instead arise from other sources of modulation, or indeed, may not even reflect active modulation. Contrary to these possibilities, however, DCM analyses robustly confirmed that the right MFG modulates the MTL during suppression (an influence likely achieved polysynaptically) (see, e.g., Anderson et al., 2016). Specifically, these analyses favored models wherein right MFG modulated all three MTL regions (parahippocampus, hippocampus, and amygdala) beyond simpler models that excluded the amygdala as a target. This superiority arose even though model selection in DCM penalizes for unnecessary model complexity (Stephan et al., 2007). Although the functional significance of amygdala is not limited to processing aversive information (e.g., Bonnet et al., 2015; Weymar and Schwabe, 2016), our findings nonetheless indicate that reduced amygdala activity when people suppressed unpleasant images in this and prior studies (Depue et al., 2007) likely reflected inhibition in service of affect regulation rather than simply being a downstream effect of memory control.

### Reactive control and supramodal inhibition

The current data support a tight connection between intrusions and inhibitory modulation of the MTL. Indeed, intrusions triggered stronger downregulation in all three regions that we studied, replicating findings in the hippocampus when people suppress neutral word pairs (Levy and Anderson, 2012) or scenes (Benoit et al., 2015), and extending this pattern to the amygdala when people suppress negative scenes. Moreover, a frontally mediated inhibitory signal appeared to drive these effects, as reflected in greater negative coupling between the right MFG and MTL subregions during intrusions. Indeed, Non-Intrusions generated no reliable evidence of negative coupling. These findings suggest that inhibitory control mechanisms were engaged reactively to disrupt momentary awareness of unwanted memories.

Our evidence for parallel modulation of the hippocampus and amygdala echoes findings observed when people suppress visual object memories. Gagnepain et al. (2014) found that suppressing such memories modulated not only the hippocampus, but also fusiform cortex regions involved in object perception. Similarly, we found downregulation in the parahippocampus and the amygdala, previously associated with scene and emotion reactivation, respectively (Smith et al., 2006; Staresina et al., 2012). Together, these findings suggest that suppression modulates cortical sites specific to the content being controlled, and that modulation is triggered by intrusion-related reinstatement of activity that is then rapidly truncated (Hellerstedt et al., 2016). Based on this “reinstatement principle” (Gagnepain et al., 2014; Hu et al., 2017), suppression should downregulate amygdala activity and play an affect-regulation function whenever the memories being suppressed elicit emotional responses reinstated by reminders, prompting the targeting of that structure for control.

Recent evidence suggests the existence of a supramodal inhibitory control process spanning motor, memory, and emotion stopping, within the right anterior MFG (Depue et al., 2016). The present findings establish novel support for this claim. The claims by Depue et al. (2016) were based on a conjunction analysis performed on the inhibition contrast for three stopping tasks spanning these domains. The colocalized right MFG activations suggest that components of the stopping mechanisms engaged in these tasks are domain general. In this study, our PLS analysis identified individual voxels that jointly predicted both affect suppression scores and intrusion regulation. Strikingly, the anterior middle frontal gyrus region identified here closely resembles the supramodal region identified by the conjunction analysis of Depue et al. (2016). Our study also adds evidence that this MFG region is effectively connected to both the hippocampus and the amygdala when people suppress unpleasant scenes, consistent with a single mechanism supporting memory control and affect regulation.

The foregoing shared mechanism hypothesis is consistent with the observation that the likely pathways supporting the regulation of the amygdala and the hippocampus transit through common relays, including the ventral portion of the PFC and the dorsal portion of the anterior cingulate cortex (Thayer et al., 2009, 2012; Ray and Zald, 2012; Anderson et al., 2016). Nevertheless, aspects of the network implementing each ability surely differ. The MFG has been proposed to reactively interrupt ongoing retrieval in the hippocampus through the thalamus (Anderson et al., 2016) or to regulate an anticipated emotional state (Etkin et al., 2015) via the ventral portion of the lateral PFC (i.e., inferior frontal gyrus), which provides direct input to the amygdala (Ray and Zald, 2012). Thus, even if common control processes in the right MFG mediate affect regulation and mnemonic control, the targets of modulation and the pathways achieving it must diverge.

### Implications

One finding with important implications is the observation of substantial individual differences in the affective consequences of retrieval suppression. Although suppression reduced amygdala activation when unpleasant scenes intruded, our participants, as a whole, did not show diminished negative affect for suppressed items. However, whereas participants with effective memory control (i.e., fewer intrusions) showed affect suppression, others with poor memory control (more intrusions) exhibited the opposite pattern: increased negative affect for suppressed compared with baseline scenes. These differences suggest that the overall null effect mixes people for whom suppression did and did not work well. Corroborating this interpretation, we found that the better that individuals were at controlling intrusions, the stronger was their engagement of inhibitory control, as reflected by (1) greater right MFG engagement during intrusions and (2) larger intrusion-related downregulations in the hippocampus and amygdala. Critically, effective connectivity analyses established that controlling intrusions triggered greater negative coupling between the MFG and these MTL regions, confirming the involvement of inhibitory modulation. This observation suggests that individual differences in inhibitory control may play a critical role in whether suppressing intrusions is an effective coping strategy. Consistent with this, increasing memory inhibition ability (indexed by suppression-induced forgetting or, electrophysiologically, by the suppression-related N2 component) predicts reduced distress from intrusive memories during the week following viewing of a traumatic film (Streb et al., 2016).

We expected that suppressing negative scenes would be harder because we assumed that emotion renders unpleasant memories especially intrusive. Our findings did not confirm this expectation. Neither overall intrusion frequency nor the decline in intrusions over suppressions varied with valence; if anything, participants reported fewer intrusions for negative scenes (Fig. 2). Moreover, inhibitory control regions were not reliably more engaged for negative scenes, nor were MTL regions more suppressed. It is unclear why valence did not affect intrusion control. One likely possibility is that our training carefully matched learning for neutral and negative associations, possibly eliminating differences in encoding strength that usually favor emotional materials. Indeed, failure to match encoding quality may partially account for why valence effects on retrieval suppression have proven inconsistent, with some studies showing greater suppression for negative content (Depue et al., 2006; Lambert et al., 2010) and others finding less (Butler and James, 2010; Nørby et al., 2010; Chen et al., 2012) or no difference (Murray et al., 2011; van Schie et al., 2013; Murray et al., 2015).

By clarifying how retrieval suppression contributes to affect regulation, the present findings may offer insights into the mechanisms underlying intrusive symptoms in psychiatric disorders. We found that successfully suppressing negative intrusions also regulates emotional responses to the intruding memory; this likely reflects the suppression of affective traces. If so, diminished suppression ability should render people vulnerable not only to recurring intrusions but also to persisting emotional responses that cascade into further distress. Indeed, difficulty controlling upsetting images frequently initiates psychopathological symptoms in disorders, including post-traumatic stress and obsessive-compulsive disorders (Brewin et al., 2010). Moreover, compromised suppression-induced forgetting occurs in people with post-traumatic stress disorder (Catarino et al., 2015), high ruminators (e.g., Fawcett et al., 2015), high trait anxiety (Marzi et al., 2014), and low resistance to stress (Lemoult et al., 2010). Some evidence even indicates that attempts to suppress thoughts in such populations can be counterproductive in some circumstances (see, e.g., Davies and Clark, 1998; Shipherd and Beck, 2005). If deficient retrieval suppression contributes to such adverse outcomes, this raises the hope that interventions focused on training the mechanisms identified here could, in principle, reduce intrusions while dampening negative affect linked to suppressed images.
